# Factors associated with preschool workers’ willingness to continue working

**DOI:** 10.1097/MD.0000000000013530

**Published:** 2018-12-10

**Authors:** Jun Tayama, Yuri Yoshida, Ryoichiro Iwanaga, Akiko Tokunaga, Goro Tanaka, Akira Imamura, Akihito Shimazu, Susumu Shirabe

**Affiliations:** a Graduate School of Education; b Center for Child Mental Health Care and Education; c Faculty of Education, Nagasaki University; d Department of Psychiatric Rehabilitation Science, Nagasaki University Graduate School of Biomedical Sciences; e Department of Occupational Therapy; f Department of Neuropsychiatry, Nagasaki University Graduate School of Biomedical Sciences, Nagasaki; g College of Liberal Arts and Sciences, Kitasato University; h Organization of Rural Medicine and Resident Education, Nagasaki University Hospital, Japan.

**Keywords:** mental health, preschool worker, social support, turnover intention, willingness to continue working, work engagement

## Abstract

In industrialized countries, the turnover rate of preschool workers is extremely high and now represents a social problem. Consequently, it has become important to promote stable early care and educational environments for this population. Several factors related to working environments are known to affect turnover intention; however, the specific factors related to turnover intention among preschool workers have not yet been identified. Considering this, the objective of the present study was to determine factors associated with preschool workers’ willingness to continue working.

The participants of this study were 1137 preschool workers based in Nagasaki Prefecture, Japan. Multivariate logistic regression analysis was used to analyze the participants’ data, with willingness to continue working for 5 years or more set as the dependent variable.

Analysis of the results for all subjects clearly showed that male sex, older age, good mental health, high number of social supports, and good work engagement have a positive effect on willingness to continue working. Further, stratifying the participants in terms of age revealed that for preschool workers under 39 years, male sex, good mental health, high number of social supports, and good work engagement act positively in regard to willingness to continue working. Meanwhile, for those over 40 years, it was found that permanent employment and good work engagement act positively in this regard. Thus, work engagement was the only common factor between preschool workers under 39 and over 40.

The findings of this cross-sectional study demonstrate that the factors associated with willingness to continue working among preschool workers differ between younger and older professionals. These findings underline the importance of considering age categories when attempting to maximize such professionals’ willingness to continue working.

## 1 Introduction

In industrialized countries, the turnover rate of preschool workers is extremely high, and now represents a serious social problem;^[1,2]^ in particular, this has become a major issue for childcare-related organizations. For instance, in a 2006 survey of 308 people engaged in child-welfare services in Sweden, 48% reported intending to leave their positions.^[1]^ Many preschool workers also report having health problems, particularly musculoskeletal complaints.^[3–5]^ Further, regarding the mental health aspects of preschool workers, low satisfaction in work is common among this population.^[2,6]^ Thus, preschool work is a profession that induces a high mental and physical burden. However, it has been determined that appropriate rewards are important factors influencing these professionals’ willingness to continue working.^[1,7]^ Further, for such workers, it is important to promote more stable early care and educational environments.

Several occupational factors are known to affect turnover intention. For example, the job demands-resources model, which is commonly used to measure turnover intention, suggests that strain (a contributor to turnover intention) is a response to an imbalance between job demands and job resources.^[8,9]^ Social support is also a job factor, and supervisor support and organizational support have been determined to contribute to reductions in turnover intention;^[10]^ conversely, social exclusion at work has been found to increase turnover intention.^[11]^ Effort-reward imbalance (ERI) in work is also considered to be related to higher turnover intention, mainly due to its association with burnout;^[5,12]^ for example, in a cross-sectional study of 436 elementary school teachers in China, logistic regression analysis revealed that ERI is an independent risk factor for burnout.^[12]^

As of 2018, approximately 10 studies have investigated turnover among preschool workers. These studies have generally focused on the following 4 areas: pain,^[3–5]^ ERI,^[4,5]^ workplace environment,^[1,7,13]^ and job satisfaction.^[2,6]^ Regarding pain, as mentioned, many preschool workers develop skeletal muscle problems.^[3]^ The reason for this is that the tasks of preschool workers include performing troublesome work, lifting heavy objects, and maintaining a static working posture for long periods of time;^[3]^ further, a longitudinal study of 199 preschool workers in Hamburg revealed that ERI causes musculoskeletal symptoms and burnout. Thus, it is possible that the occurrence of health problems particular to preschool workers can cause a desire to leave their jobs. Regarding workplace environments, it has been found that remuneration affects willingness to continue working,^[1,7]^ and that the type of treatment received in work environments influences psychological happiness.^[13]^ Finally, regarding job satisfaction, a study of 525 preschool workers showed that turnover intention is low when workplace satisfaction is high.^[6]^

In recent years, research into the work engagement of workers has been rapidly gaining popularity.^[14–16]^ Consequently, it has been found that strong work engagement is related to high work ability,^[17]^ low psychological distress,^[18]^ and low burnout.^[14]^ For example, a study of 403 Finnish firefighters revealed that work engagement is related to work ability;^[17]^ meanwhile, with regard to psychological distress, cross-sectional studies of 894 Japanese manufacturing company staff showed that work engagement increases and psychological distress lessens when there are many structural job resources available.^[18]^

In fact, many studies have reported that high work engagement also lowers turnover intention.^[16,19,20]^ For example, Forbes et al^[21]^ conducted a cross-sectional study on job resources and turnover intention in regard to 231 dental nurses in Scotland, finding that the most important job resource is a good working relationship; similar results were found in a study of 215 Portuguese nursing staff.^[22]^

Despite the above findings, no previous study has focused on the relationship between work engagement and turnover intention in regard to preschool workers. Further, for preschool workers, there may also be factors other than work engagement that is related to turnover intention, and this also requires investigation. Considering this, it is clearly necessary to study the factors associated with preschool workers’ willingness to continue in their posts, and this represents the focus of the present study.

## 2 Methods

### 2.1 Subjects

The participants were preschool workers who had worked in Nagasaki Prefecture, Japan, or a similar institution between April 2017 and December 2017. Overall, 1,137 Japanese preschool workers were recruited and, of these, 214 individuals failed to fully answer the questionnaire. Thus, the final sample consisted of 923 preschool workers. Of this final sample, 35% were aged in their 20 seconds, 27% were in their 30 seconds, 22% were in their 40 seconds, and 16% were over 50. Women accounted for 98% of the subjects. The study protocol was approved by the Ethics Committee of Nagasaki University (approval no. 17060864), and the study was conducted in accordance with the ethical standards of the 1964 Declaration of Helsinki and its later amendments. All participants provided written informed consent before study participation. With regard to data availability, all relevant data are included within the paper.

### 2.2 Procedure

Participants completed the study questionnaire in their own workplaces. The questionnaire, which required approximately 10 minutes to complete, collected data regarding sex, age, occupation, form of employment, willingness to continue working in their present position for 5 years or more, mental health, number of social supports available, and work engagement. Using these data, the participants were divided into 2 groups according to their willingness to continue working in their present positions for 5 years or more (“yes” or “no”). They were also divided into 2 groups based on their form of employment (permanent employee, contract employee). Then, their occupations were classified into one of the following 4 categories:

(1) kindergarten teacher,
(2) childcare teacher,
(3) childcare-provider,
(4) and other.

Meanwhile, the participants were also classified in terms of age into one of the following 4 categories:

(1) 20 to 29,
(2) 30 to 39,
(3) 40 to 49,
(4) ≥50.

Next, considering mental health, which was assessed using the K6,^[23]^ the participants were classified into one of the following 2 categories based on their scores: K6 <13 or K6 ≥13; K6 ≥13 indicated the presence of psychological distress. For the amount of social supports available, the participants were classified into one of the following 4 categories:

(1) 0 to 1,
(2) 2 to 3,
(3) 4 to 5,
(4) ≥6.

Finally, for work engagement, which was assessed using the Utrecht Work Engagement Scale (UWES; high UWES scores indicate high work engagement),^[24]^ the participants were classified into one of the following 4 categories based on their scores:

(1) Quartile 1,
(2) Quartile 2,
(3) Quartile 3, and
(4) Quartile 4.

### 2.3 Measures

#### 2.3.1 Work engagement

Work engagement was operationalized using UWES, a self-report instrument that includes 3 dimensions, comprising 17 items altogether:^[14]^ vigor (6 items), dedication (5 items), and absorption (6 items). All items are scored using a 7-point Likert scale, which ranges from 0 (“never”) to 6 (“always”). Inter-correlations and internal consistencies (Cronbach's α on the diagonal) of the 3 scales of the original version of the UWES (vigor, dedication, and absorption) have previously been deemed to be sufficient.^[15]^

#### 2.3.2 Social support questionnaire (SSQ)

The SSQ assesses the perceived availability of and satisfaction with social support, which is usually defined as the existence or availability of people on whom we can rely.^[25,26]^ The internal consistency, factor validity, and construct validity of the Japanese version of the SSQ have previously been determined to be high.^[27]^ The short version of the SSQ consists of 12 items, and each item has 2 parts. Six of the items measure the perceived amount of social support, and the other 6 measure satisfaction with social support. The items that measure satisfaction with social support are rated using a 6-point Likert scale (1 = “very dissatisfied” to 6 = “very satisfied”). The average scores for the 2 domains are then calculated. The Cronbach's alpha coefficient for the SSQ Number subscale was .91, and that for the SSQ Satisfaction subscale was .94 (Furukawa et al, 1999).^[27]^ In this study, we only used the amount subscale. Since satisfaction with social support (as indexed by the SSQ) is related to social desirability and neuroticism,^[25]^ it was not assessed in the present study.

#### 2.3.3 Mental health

The 6-item K6 was used to evaluate the presence/absence of psychological distress among the subjects.^[23]^ The K6 was developed to identify persons who are at a risk of developing mental states such as depression and anxiety.^[28]^ The total K6 score can range from 0 to 24, and patients with scores of 13 or greater are categorized as suffering from psychological distress.

### 2.4 Data analyses

First, using the data for all of the subjects, multivariate logistic regression analysis was performed, using willingness to continue working for 5 years or more as the dependent variable. Next, another multivariate logistic regression analysis was performed, again using willingness to continue working for 5 years or more as the dependent variable, but with the results stratified into those for participants under 39 years old and those for participants over 40 years old. Both crude and adjusted odds ratios (ORs) and 95% confidence intervals (CIs) were calculated. In calculating multivariate odds, sex, age, occupation, form of employment, willingness to continue working for 5 years or more, mental health, and number of social supports available were set as covariates. JMP ver. 10.0 software was used for all statistical analyses.

## 3 Results

### 3.1 Univariate and multivariate analysis of the associations, for all participants, between willingness to continue working for 5 years or more and demographics, social support, and work engagement

Of the analyzed population, the crude percentage showing willingness to continue working in their current positions for 5 years or more was 41.6% (384/923; 95% CI [38.5, 44.8]). Univariate analysis showed that the following factors were significantly associated with higher willingness to continue working for 5 years or more: male sex, older age, good mental health (K6 <13), high number of social supports available, and high work engagement (Table 1). After adjusting for covariates, these results remained unchanged.

**Table 1:**
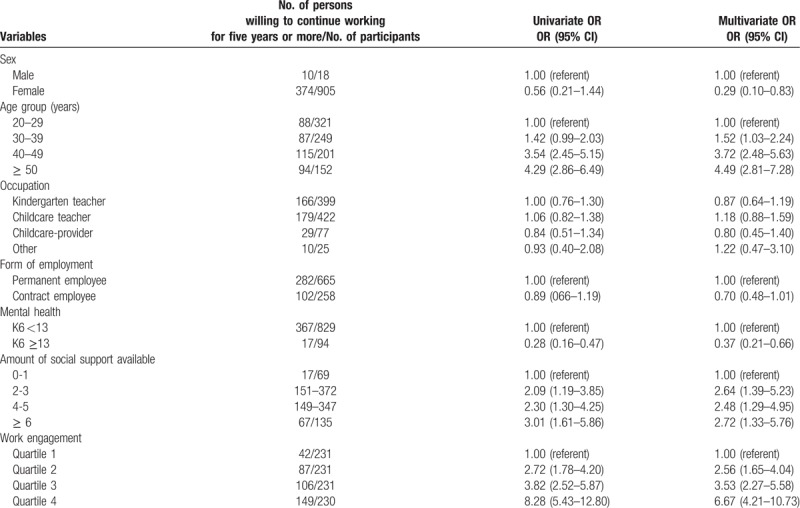
Univariate and multivariate analysis of the associations, for all participants, between willingness to continue working for 5 years or more and demographics, social support, and work engagement.

The proportion of those willing to continue working for 5 years or more increased with age (Table 1). In regard to amount of social support, if the number of social supports was 2 or more, the odds ratio of those willing to continue working for 5 years or more was 2.00 or more when compared with participants for whom the number of social supports available was 0 to 1 (Table 1). For work engagement, the odds ratio of those willing to continue working for 5 years or more increased in accordance with increased work engagement (Table 1).

### 3.2 Univariate and multivariate analysis of the associations, for participants under 39 years of age, between willingness to continue working for 5 years or more and demographics, social support, and work engagement

Of the participants under 39 years of age, the crude percentage showing willingness to continue working for 5 years or more was 30.7% (175/570; 95% CI [27.1, 34.6]). Univariate analysis showed that, among this population, the following factors were significantly associated with a higher willingness to continue working for 5 years or more: good mental health (K6 <13), high number of social supports, and high work engagement (Table 2). After adjusting for covariates, male sex was also determined to be significantly associated with a higher willingness to continue working for 5 years or more; the other results were unchanged.

**Table 2:**
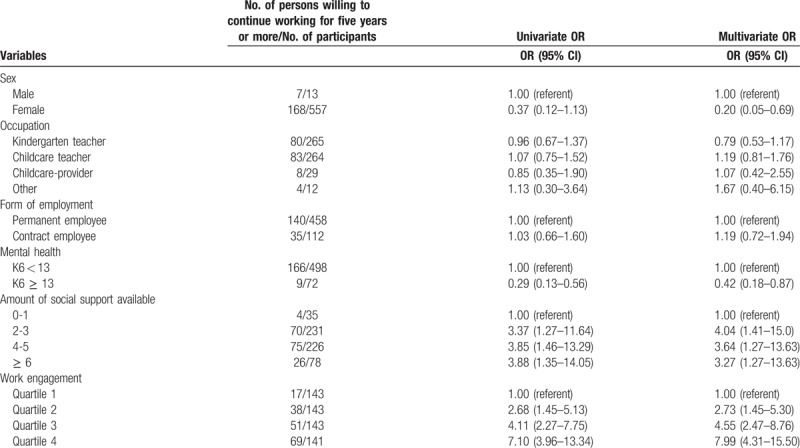
Univariate and multivariate analysis of the associations, for participants under 39 years of age, between willingness to continue working for 5 years or more and demographics, social support, and work engagement.

Regarding the number of social supports, for cases where the number of social supports was 2 or more, the odds ratio of those willing to continue working for 5 years or more was 3.00 or more when compared with those with 0 to 1 social supports (Table 2). For work engagement, the odds ratio of those willing to continue working for 5 years or more increased in accordance with increased work engagement (Table 2).

### 3.3 Univariate and multivariate analysis of the associations, for participants over 40 years of age, between willingness to continue working for 5 years or more and demographics, social support, and work engagement

For the participants over 40 years of age, the crude percentage showing willingness to continue working for 5 years or more was 59.2% (209/353; 95% CI [54.0, 64.2]). Univariate analysis showed that the following factors were significantly associated with a higher willingness to continue working for 5 years or more: being a permanent employee (vs a contract employee), good mental health (K6 <13), high number of social supports, and high work engagement (Table 3). After adjusting for covariates, the results for permanent employees and work engagement did not change; however, mental health (K6 <13) and number of social supports were found to be irrelevant regarding willingness to continue working for 5 years or more.

**Table 3:**
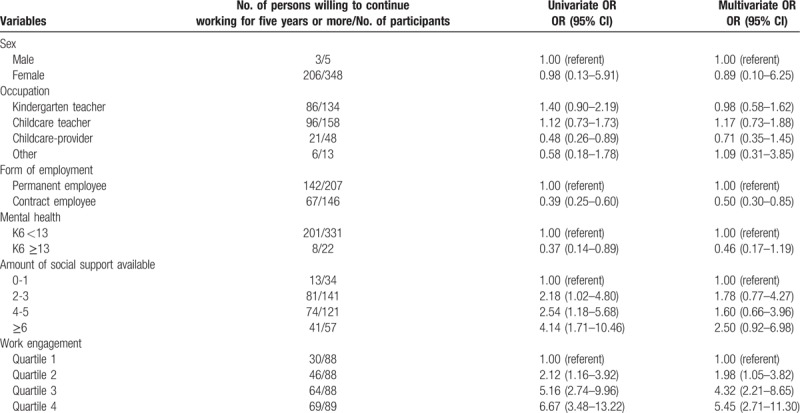
Univariate and multivariate analysis of the associations, for participants over 40 years of age, between willingness to continue working for 5 years or more and demographics, social support, and work engagement.

For work engagement, the odds ratio of those willing to continue working for 5 years or more increased in accordance with increased work engagement (Table 3).

## 4 Discussion

Analysis of the results for all subjects showed that male sex, older age, good mental health, high number of social supports, and high work engagement have a positive effect on willingness to continue working. Applying stratification of age revealed that, among preschool workers who are under 39 years of age, male sex, good mental health, high number of social supports, and high work engagement have a positive effect on willingness to continue working. Meanwhile, for preschool workers over 40, permanent employment and high work engagement were determined to act positively in regard to willingness to continue working. Work engagement was the only common factor that increased willingness to continue working for both under 39 seconds and over 40 seconds.

Several previous studies that examined the relationship between work engagement and turnover intention have reported that high work engagement is associated with low turnover intention.^[19–21,29]^ As mentioned above, a cross-sectional survey of 231 dental nurses in Scotland found that a good working relationship inhibits turnover intention^[21]^; the study also found that, for dental nurses, work engagement shows a strong negative correlation with turnover intention. Meanwhile, in a study of 316 nurses based in Ibero-American countries, it was revealed that both burnout and work engagement affect turnover intention.^[29]^ Other studies have shown that job demand and job control affect work engagement,^[30]^ and that poor workplace environments may lower work engagement^[7]^ and may increase turnover intention. Meanwhile, conversely, a good working environment has been shown to lower turnover intention.^[1]^ In the present research, it was shown that high work engagement benefits willingness to continue working. It is possible that the preceding factor of a good workplace creates high work engagement and, in turn, willingness to continue working manifests as a subsequent factor.

For young preschool workers’ willingness to continue working, social support was determined to be important. However, social support was found not to be important for preschool workers over 40. Other studies have shown that, for young people, social support may contribute to prevention of turnover intention. In a cross-sectional study of 215 Portuguese nursing staff with an average age (± SD) of 39 (± 9) years, cross-sectional research revealed that social support among peers increases work engagement and decreases turnover intention.^[22]^

Further, mental health was also found to be important for young preschool workers’ willingness to continue working; however, mental health was not important for preschool workers over the age of 40. As with social support for young men, measures to maintain mental health may contribute to the prevention of turnover intention. Burnout has previously been found to increase turnover intention,^[29,31]^ and it is also known that burnout is likely to occur due to low work engagement.^[29,30,32–34]^ In other words, when work engagement negatively affects mental health, turnover intention may consequently increase. Therefore, the introduction of mental care and self-care for young preschool workers may lead to the suppression of turnover intention.

For the participants over 40, willingness to continue working was determined to be linked to permanent employment. However, permanent employment was not important for the under-39 seconds. Good rewards and welfare are known to contribute to the suppression of turnover intention;^[1,7,13]^ thus, for individuals over the age of 40, there is a possibility that good treatment in regard to employment may lead to lower job-separation desire.

There were important limitations to this study. All of the participants were sourced from a single prefecture; specifically, from a medium-sized city on the island of Kyushu, in western Japan. Thus, it is unclear whether the results can be extrapolated to Japanese preschool workers in general. In the future, participants should be sourced from several prefectures in Japan.

In conclusion, the findings of this cross-sectional study demonstrate that the factors associated with preschool workers’ willingness to continue working differ between younger and older workers. These findings underline the importance of considering age categories when attempting to maximize preschool workers’ willingness to continue working.

## Acknowledgments

The authors would like to express their appreciation to the participants of this study. This study was supported by the Center for Child Mental Health Care and Education, and Nagasaki University.

## Author contributions

**Conceptualization:** Jun Tayama, Ryoichiro Iwanaga, Akiko Tokunaga, Goro Tanaka, Akira Imamura, Akihito Shimazu, Susumu Shirabe.

**Data curation:** Akiko Tokunaga.

**Formal analysis:** Jun Tayama.

**Investigation:** Ryoichiro Iwanaga, Akiko Tokunaga.

**Methodology:** Jun Tayama, Akihito Shimazu.

**Resources:** Ryoichiro Iwanaga.

**Supervision:** Yuri Yoshida, Goro Tanaka, Akira Imamura, Akihito Shimazu, Susumu Shirabe.

**Writing – original draft:** Jun Tayama.
